# Recombination-aware phylogenetic analysis sheds light on the evolutionary origin of SARS-CoV-2

**DOI:** 10.1038/s41598-023-50952-1

**Published:** 2024-01-04

**Authors:** Luis Roger Esquivel Gomez, Ariane Weber, Arthur Kocher, Denise Kühnert

**Affiliations:** 1 Transmission, Infection, Diversification and Evolution Group (tide), Max Planck Institute of Geoanthropology (Formerly MPI for the Science of Human History), Jena, Germany; 2 Department of Archaeogenetics, Max Planck Institute for Evolutionary Anthropology, Leipzig, Germany; 3 Phylogenomics Unit, Center for Artificial Intelligence in Public Health Research, Robert Koch Institute, Wildau, Germany

**Keywords:** Phylogenetics, SARS-CoV-2

## Abstract

SARS-CoV-2 can infect human cells through the recognition of the human angiotensin-converting enzyme 2 receptor. This affinity is given by six amino acid residues located in the variable loop of the receptor binding domain (RBD) within the Spike protein. Genetic recombination involving bat and pangolin *Sarbecoviruses*, and natural selection have been proposed as possible explanations for the acquisition of the variable loop and these amino acid residues. In this study we employed Bayesian phylogenetics to jointly reconstruct the phylogeny of the RBD among human, bat and pangolin *Sarbecoviruses* and detect recombination events affecting this region of the genome. A recombination event involving RaTG13, the closest relative of SARS-CoV-2 that lacks five of the six residues, and an unsampled *Sarbecovirus* lineage was detected. This result suggests that the variable loop of the RBD didn’t have a recombinant origin and the key amino acid residues were likely present in the common ancestor of SARS-CoV-2 and RaTG13, with the latter losing five of them probably as the result of recombination.

## Introduction

At the end of the year 2019, human cases of a new respiratory disease were detected in Wuhan, China^1,2^. It was determined that the infectious agent was a new RNA virus member of the Family Coronaviridae, named Severe acute respiratory syndrome coronavirus 2 or SARS-CoV-2^3^. The virus spread worldwide during the next months, and cases have been reported in more than 200 countries. Over 600 million infections and more than 6.9 million deaths have been attributed to this virus as of November 2023 (https://covid19.who.int/). SARS-CoV-2 is the third coronavirus capable of inducing severe respiratory diseases in humans to emerge in the last 18 years, after the severe acute respiratory syndrome coronavirus (SARS-CoV-1), detected in China in 2002^4^, and the Middle East respiratory syndrome coronavirus (MERS-CoV) identified in the year 2012 in Saudi Arabia^5^.

**Figure 1:**
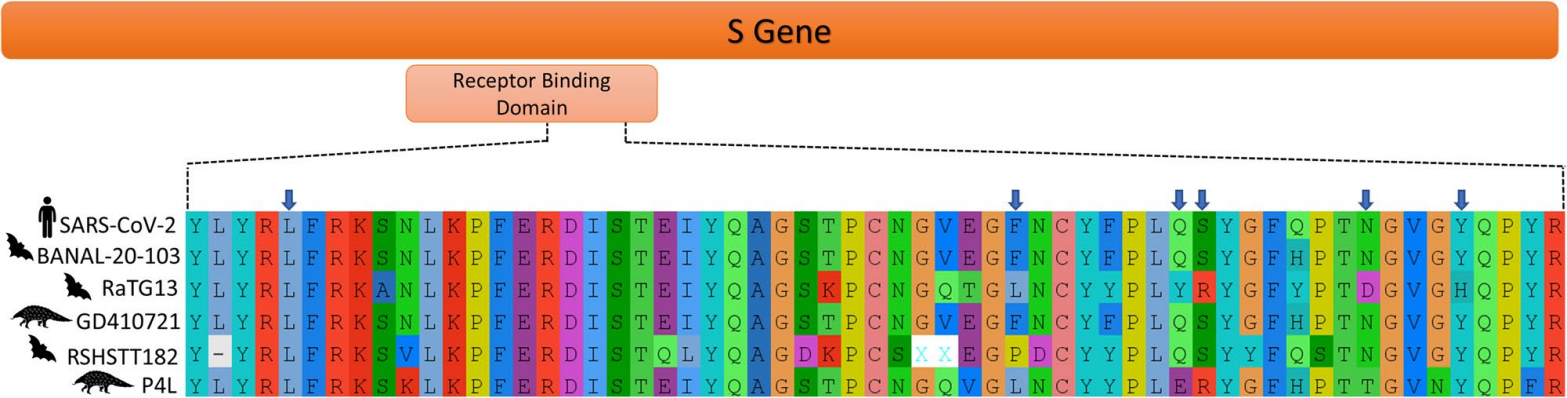
Amino acid residues present in the variable loop of the receptor binding domain of SARS-CoV-2 and related viruses. Amino acid residues important for the recognition of hACE2 receptor in SARS-CoV-2 are indicated with the blue arrows.

SARS-CoV-2, along with SARS-CoV-1, is a member of the subgenus *Sarbecovirus*, and has a positive sense single-stranded RNA genome with a length of approximately 30 kb, that encodes four major structural proteins: spike, envelope, membrane, and nucleocapsid, encoded by the S, E, M and N genes respectively^6^. The spike protein has a central role in cell infection and pathogenesis, since it mediates the recognition of cellular receptors and the binding of the viral and cell membranes, a process that ultimately leads to the entry of the virus into the cell^7^. The spike protein contains a receptor binding domain (RBD), which gives the virus an affinity for the angiotensin-converting enzyme 2 (hACE2), a human cell receptor also used by SARS-CoV-1 for the binding process^1,8^. The RBD of SARS-CoV-2 is characterized by six contact amino acid residues that are essential for the binding to the hACE2 receptors^9^, and are present in a region known as the variable loop^10^ (Fig. 1). The RBD is the most variable part of the genome among coronaviruses and has an important role in determining the host range of each viral species^9,11^.

The evolutionary origin of SARS-CoV-2 remains unclear so far. Analyses of full genome sequences indicate that the closest known relatives of this virus are the bat *Sarbecoviruses* RmYN02 and RaTG13^2,12^, which suggests an emergence in humans following a spillover from bats directly or through an intermediate host. However, *Sarbecoviruses*, like other coronaviruses, are highly recombinant^10,13^ and the examination of the SARS-CoV-2 RBD sequence has suggested more complex scenarios for its origin involving genetic recombination. Indeed, while bat viruses are the closest relatives of SARS-CoV-2 considering the full genome sequences, the RBDs of SARS-CoV-2 and GD410721, a *Sarbecovirus* isolated from Malayan pangolins (*Manis javanica*), are highly similar, and share the six amino acid residues that confer the affinity for hACE2 receptors^14,15^.

**Figure 2:**
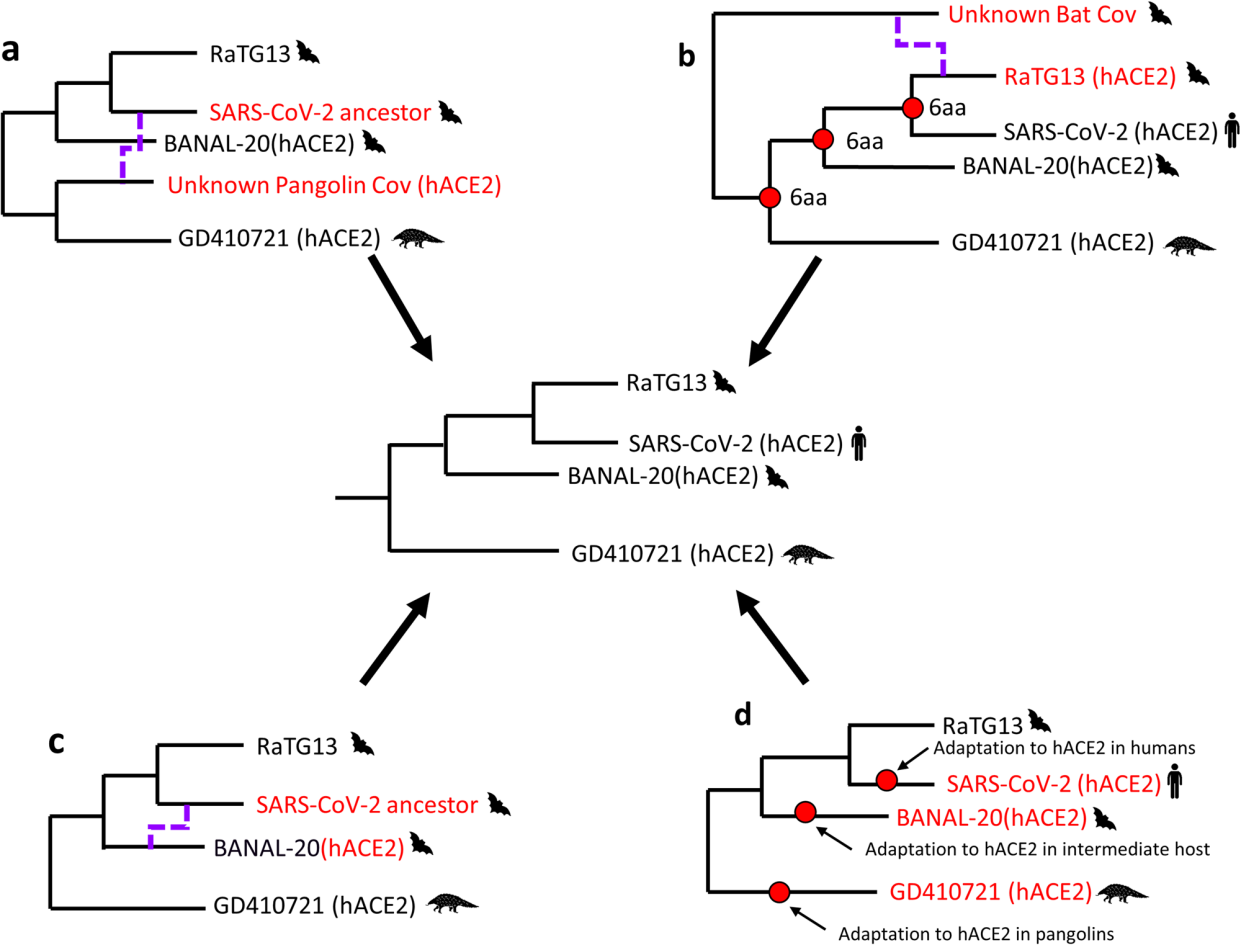
Four hypotheses to explain the presence/absence of the six contact residues in the RBD of human, bat and pangolin viruses that confer a high affinity for human ACE2 receptors (hACE2). (**a**) SARS-CoV-2 acquired the six amino acid residues after a recombination event (dashed line) between unknown bat and pangolin viruses (in red). (**b**) RaTG13 lost five of the six amino acid residues after a recombination event with an unknown Sarbecovirus. (**c**) The amino acid residues were acquired from a bat Sarbecovirus. (**d**) The six amino acid residues were acquired as a result of adaptation to recognize the same type of cellular receptors. The central tree represents the expected topology for the S gene phylogeny.

Hence, three of the four main hypotheses for the origin of the variable loop of SARS-CoV-2 include recombination (Fig. 2). First, it has been postulated that SARS-CoV-2 acquired the variable loop of the RBD after a recombination event with a pangolin *Sarbecovirus* (Fig. 2a). Previous studies have employed tools for the detection of recombination breakpoints and the generation of similarity plots, to find signals of recombination events in the S gene involving SARS-CoV-2, RaTG13 and GD410721^15–17^. A second hypothesis proposed by Boni et al.^10^ suggests that the common ancestor of SARS-CoV-2, GD410721 and RaTG13 was capable of recognizing the hACE2 receptors and part of the RBD of RaTG13 was replaced through recombination with another *Sarbecovirus* missing the hACE2-specific residues (Fig. 2b). The recent discovery of *Sarbecoviruses* in different species of cave bats (BANAL-52, -103, and -236), presenting an almost identical RBD to the one from SARS-CoV-2 has motivated a third hypothesis, in which SARS-CoV-2 could have acquired the contact residues as the result of recombination events among bat *Sarbecoviruses*^18^ (Fig. 2c). Finally, the affinity for hACE2 receptors found in viruses that infect different hosts carrying the same type of receptors could also be the result of convergent evolution^9^ (Fig. 2d).

Given the potential importance of recombination in the evolutionary history of the S gene, the detection of recombination events has become a very important task in evolutionary studies of SARS-CoV-2. The presence of recombination among *Sarbecoviruses* has been explored previously with tools like SimPlot^19^, RDP4^20^, RDP5^21^ and GARD^22^. These methods allow the identification of recombinant strains and recombination breakpoints, as well as potential parental strains involved. However, they are unable to estimate Ancestral Recombination Graphs (ARGs) to represent the reticulated evolution induced by genetic recombination. This seriously limits the possibility to generate a comprehensive evolutionary picture, and does not allow to propagate phylogenetic uncertainties in the estimation of recombination events^23–25^.

The reconstruction of ARGs from sequence data is a notoriously difficult task. Nevertheless, several software packages have been developed to tackle this challenge. One example is the Bayesian approach implemented in the BEAST2 V2.6.3 package Bacter^24^. Bacter is an implementation of the ClonalOrigin model^26^ which can be used to estimate a special type of ARG referred to as Ancestral Conversion Graph (ACG). ACGs consist of a backbone bifurcating phylogeny representing the evolution of the major part of the genetic material (referred to as the “clonal frame”), together with recombination events involving donor and recipient lineages on the clonal frame (Supplementary Fig. S1 online). The method allows to estimate recombination events within a dated phylogeny together with measures of statistical support and uncertainties in the form of highest posterior density intervals (HPDIs). This allows us to date individual recombination events, which in turn provide us with an estimation of the emergence time of recombinant lineages. A different Bayesian approach to perform recombination-aware phylogenetic analyses produces recombination networks, where recombinant edges are also integrated within the phylogeny^27^. Although unlike Bacter, this approach doesn’t estimate the posterior support for the arrival point of the recombination on the recipient lineage. In this study we employed Bacter to detect recombination events within the RBD region of 45 *Sarbecovirus* genomes, with the goal of clarifying the origin of the amino acid residues located in the variable loop that give SARS-CoV-2 the high affinity to the hACE2 receptors.

## Results

### Temporal signal and model selection

The initial data set consisted of a sequence alignment of the RBD region of 111 S*arbecoviruses*, with potentially misaligned regions masked by the program Gblocks^28^. However, as a Bacter analysis is computationally demanding, a representative subsample of 45 viral sequences was selected with the program Uclust^29^. Using a Bayesian Evaluation of Temporal Signal (BETS) analysis^30^ we identified a significant level of temporal signal in our dataset (logBF = 2.09). The BEAST2 V2.6.3 package bModelTest^31^ selected as the best substitution model a modification of the General Time Reversible (GTR) model^32^ with the same rates for AC and CG substitutions and a proportion of invariant sites of 0.3. A model comparison with a Stepping-Stone sampling^33^ determined the constant coalescent as the best fitting tree prior (logBF = 2.14).

### Bacter analysis and recombination events

A summary ACG obtained with a posterior probability threshold of 0.9 didn’t detect significant recombination events involving SARS-CoV-2, RaTG13, BANAL-103 (a virus from cave bats) or GD410721 (Supplementary Fig. S2 online). While this result supports the host adaptation hypothesis, a summary ACG with a reduced posterior threshold of 0.1 detected two recombination events with overlapping recombinant regions and with arrival points, which indicate the recipient of the recombinant region, located in the terminal branch leading to RaTG13 (Supplementary Fig. S3 online). The two donor sequences are also unsampled members of the same phylogenetic clade, and one is closely related to a pangolin virus from Guangxi. Thus, it is very likely that just a single recombination event affected RaTG13, although there is a lot of uncertainty regarding the origin of the donor sequence.

**Figure 3:**
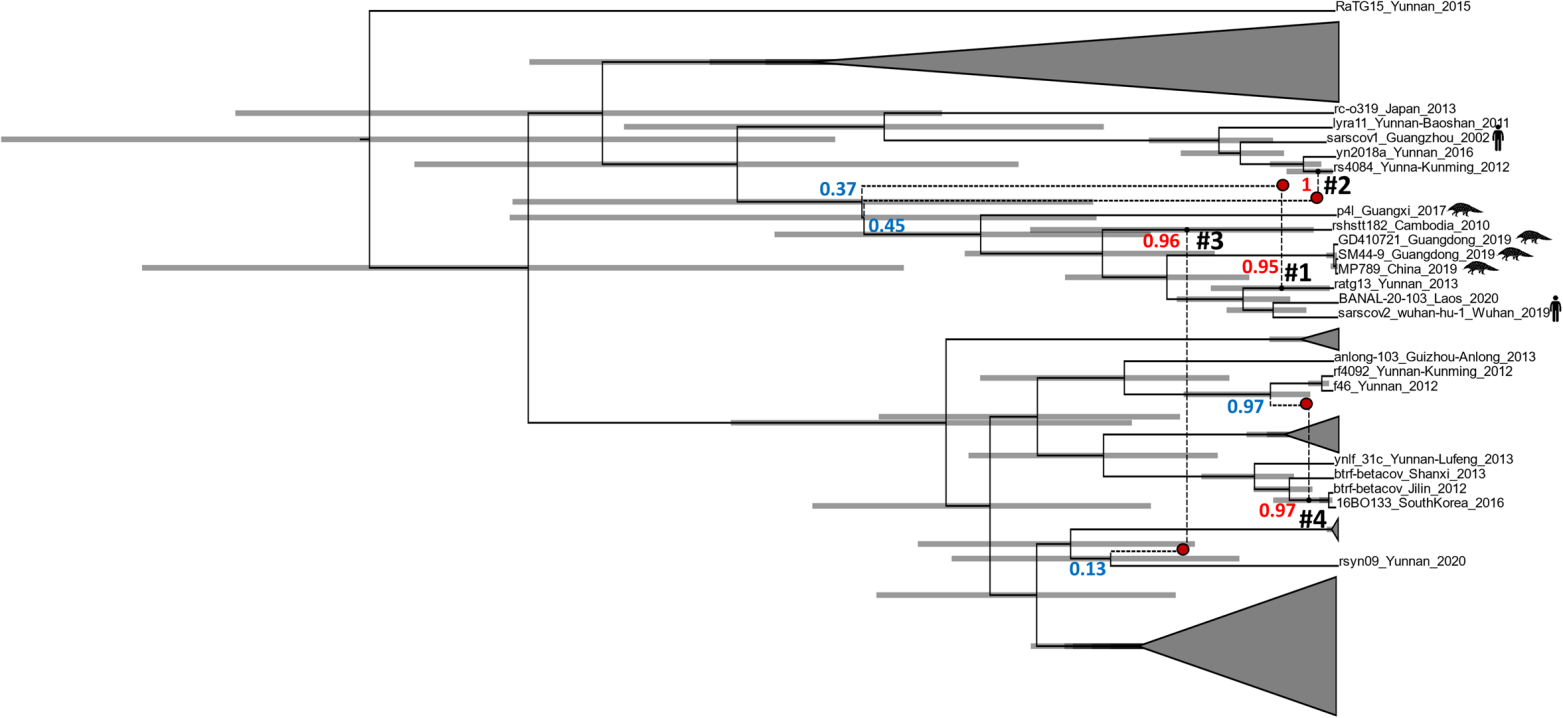
Summary ACG obtained for 45 Sarbecovirus sequences with a posterior threshold of 0.9 for recombination support and using the new implementation of ACGAnnotator. Recombination events are indicated with dashed lines. Recombination numbers mentioned in the text are in black. Posterior supports for the arrival point of each recombination event are shown in red, supports for the origin position of the donor sequences are shown in blue. Red dots represent the unsampled viruses that donated the recombinant sequences. Pangolin and human viruses are indicated by the respective species icons. The rest of the sequences were obtained from bats. Some parts of the figure are collapsed (gray triangles) to help with the visualization. 95% HPDI for the node ages are represented by the error bars.

**Figure 4:**
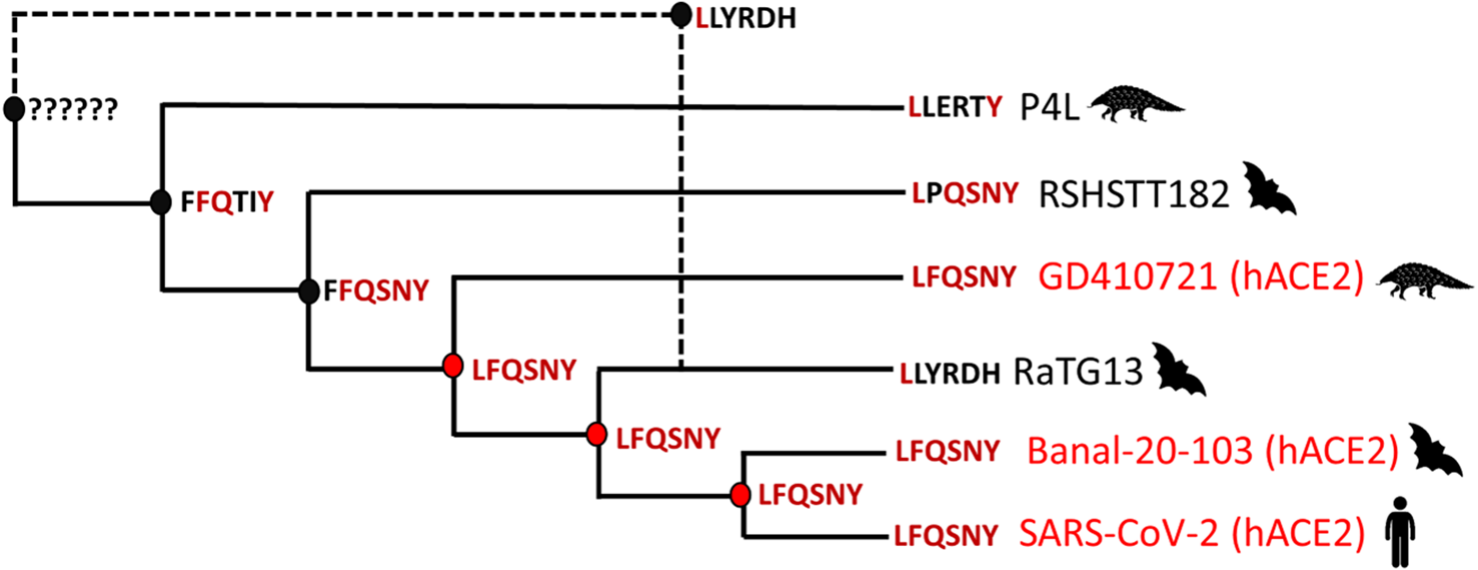
Hypothesis regarding the origin of the variable loop in SARS-CoV-2 supported by the Bacter analysis. The capital letters in bold represent the six contact amino acid residues of the variable loop present in the sequences and the ancestral states for the internal nodes obtained with the ASR analysis. In dark red are the amino acid residues present in SARS-CoV-2. Dark circles represent ancestors and the donor sequence that are not capable of binding to human cells. Red circles and names are ancestors and sequences that can bind to human cells.

The identification of low-support recombination events that only differ in the departure point, which indicates the origin of the donor of the recombinant region, suggests that the signal of one recombination is diluted when using the ACGAnnotator tool that only summarizes recombinations that depart from the same branch. To classify these recombinations as one, a new summary ACG was obtained with our new implementation of this tool. Four recombination events with a posterior probability support greater than 0.9 were recovered (Fig. 3) and as expected, one involved RaTG13. The uncertainty in the position in the clonal frame of the donor sequence that motivated the use of the new implementation of ACGAnnotator, was reflected by a low posterior support for the origin node of this virus, something also observed in three of the four recombination events (Fig. 3) In this recombination, RaTG13 received most of the second half of the RBD from an unsampled virus that belongs to the same clade as the pangolin viruses from Guangdong and Guangxi, RaTG13, SARS-CoV-2, BANAL-103 and rshstt182. This recombination occurred 84 yBP (95% HPDI: 190-13yBP) and the recombinant region, considering the HPDI of the start and end site (Table 1), includes the six contact amino acid residues of the variable loop. An ancestral sequence reconstruction (ASR) analysis done in BEAST2 showed that the most likely amino acid residues for the six contact positions of the MRCA of RaTG13, BANAL-103 and SARS-CoV-2, were the residues that confer the high affinity for the ACE2 receptor. The same result was obtained for the ancestor of those three viruses and GD410721 (Fig. 4). These results indicate that RaTG13 was indeed involved in a recombination event that likely resulted in the loss of five of the six amino acid residues.

**Table 1:** Median estimates of dates, and recombinant regions of the seven recombination events recovered by Bacter.

Recombination number	Emergence time of donor sequence yBP (95% HPDI)	Recombination date yBP (95% HPDI)	Sites	Start site (95%HPDI)	End site (95% HPDI)
1	707 (1225–364)	84 (190–13)	390–613	306–421	605–650
2	704 (1230–359)	31 (78–9)	317–640	300–400	633–650
3	338 (574–147)	225 (458–37)	0–27	0–1	21–47
4	101 (230–42)	44 (97–15)	0–55	0–0	50–61

**Figure 5:**
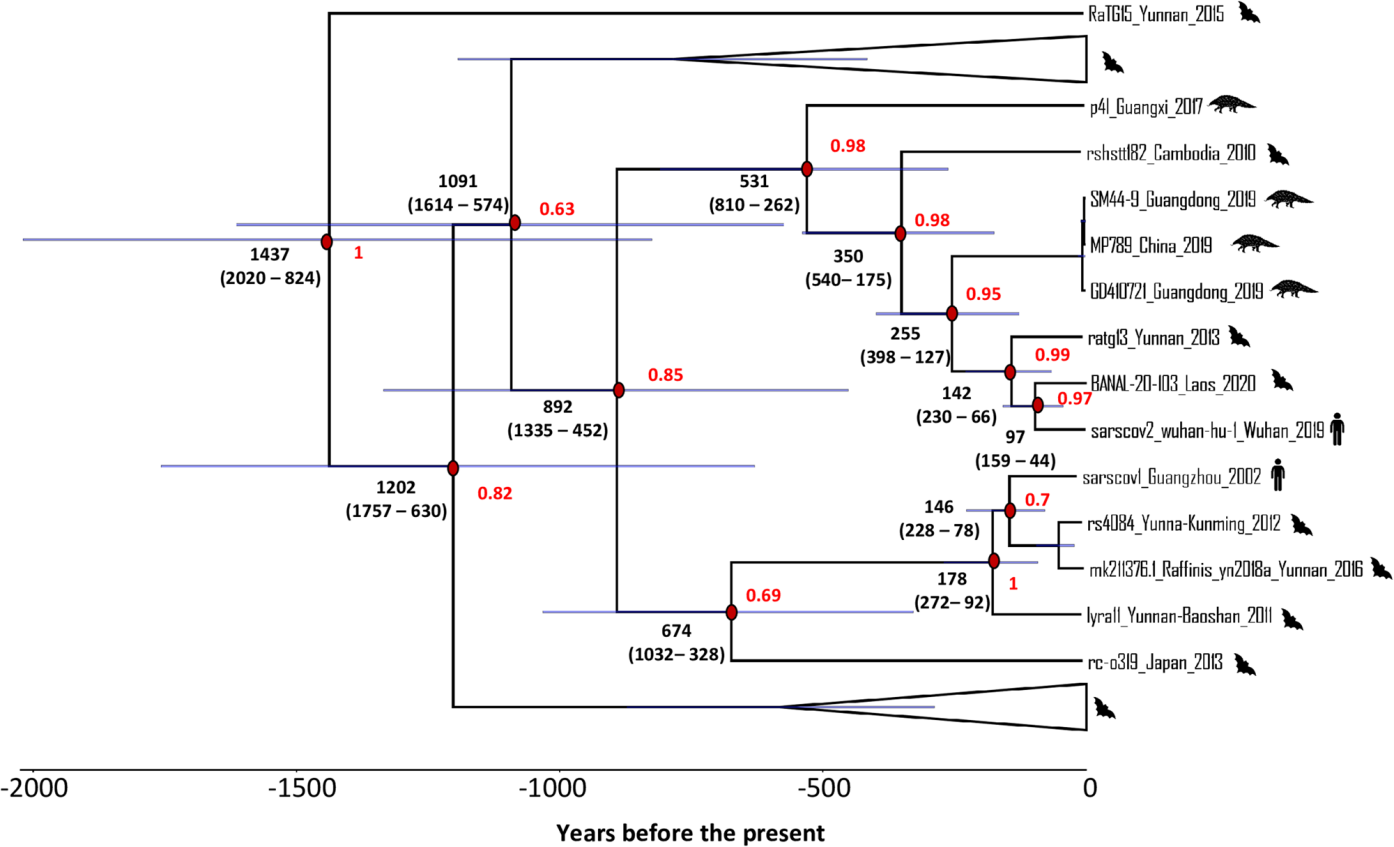
Maximum clade credibility tree representing the Clonal Frame of the RBD of 45 *Sarbecoviruses*. Median ages are shown in black with the 95% HPDI in brackets and represented by the error bars. Posterior node support is indicated with red. Recombination events have been removed and some nodes have been collapsed (triangles) to help with the visualization of the tree. Branch lengths are scaled in time.

The recombination numbers correspond to those in the text and Fig. 5. Key contact amino acid residues are located between positions 427–636.

In recombination event #2, rs4084, a virus isolated from the Chinese rufous horseshoe bat (*Rhinolophus sinicus*), also acquired most of the second half of the RBD from an unsampled taxa member of the clade conformed by SARS-CoV-2, RaTG13, BANAL-103, rshstt182 and the pangolin viruses. Unlike RaTG13, rs4084 is capable of efficiently binding to the hACE2 receptor^34^, although using a mechanism that doesn’t involve the six amino acid residues of SARS-CoV-2^35^. The different affinity for hACE2 receptor between RaTG13 and rs4084 despite both receiving second half of the RBD from closely related viruses, and the different ACE2 binding mechanisms between rs4084 and SARS-CoV-2, suggest that rs4084 received an RBD similar to the one present in RaTG13 through recombination, and afterwards it acquired the capacity to bind to ACE2 likely after jumping to different host species.

In recombination event #3, which happened 225 yBP (95% HPDI: 458-37yBP), rshstt182, a virus isolated from the Shamel's horseshoe bat (*Rhinolophus shameli*) in Cambodia, received the first 27 nucleotides of the RBD from a virus closely related to rsyn09, which was isolated in China from the lesser brown horseshoe bat (*Rhinolophus stheno*). The last recombination event occurred 44 yBP (95% HPDI: 97-15). The point of origin was detected in the ancestral branch of two bat *Sarbecoviruses* infecting the greater horseshoe bat (*Rhinolophus ferrumequinum*) and the least horseshoe bat (*Rhinolophus pusillus*). The arrival point of the recombination was located in the ancestral branch of two *Sarbecoviruses,* also infecting the greater horseshoe bat, one found in South Korea. The recombinant region consisted of the first 55 sites of the RBD.

### Recombination detection and evolutionary rate variation

Since a Bacter analysis assumes a strict molecular clock with every lineage of the clonal frame evolving at the same rate, it is possible that the recombination signals detected in the analysis were the product of different evolutionary rates among lineages and sites. In order to assess the possibility of the recombinations being false positives, evolutionary rates were estimated in BEAST2 for the recombinant region, delimited by the 95%HPDI of the start and end sites estimated by Bacter for the recombination event affecting RaTG13 (sites 300–650), and the regions of the alignment before and after the recombination breakpoints. The estimated values (Table 2), were used to simulate 10 alignments of 45 sequences assuming no recombination and different rates for the three regions of the sequences. These simulated alignments were analyzed with Bacter. Summary ACGs obtained with both implementations of ACGAnnotator showed, as expected, no substantial support for recombination events. In 9 of the 10 datasets, no recombination events were detected at all. In the remaining analysis, a single spurious recombination event with a posterior support of 0.17 (original implementation) and 0.24 (new implementation) was detected.

**Table 2:** Posterior estimates of evolutionary rates obtained for each alignment partition in a molecular dating analysis.

Partition	Mean (95% HPDI)	Median	Standard deviation (95% HPDI)
Before the first breakpoint	4.857E−4 (1.05E^−4^–9.23E^−4^)	4.599E^−4^	0.4897 (0.32–0.67)
Recombinant region	7.96E−4 (1.67E^−4^–1.52E^−3^)	7.533E^−4^	0.962 (0.73–1.2)
After the second breakpoint	5.641E−4 (1.13E^−4^–1.09E^−3^)	5.287E^−4^	0.218 (5.47E^−10^–0.52)

### Molecular dating of the RBD

**Figure 6:**
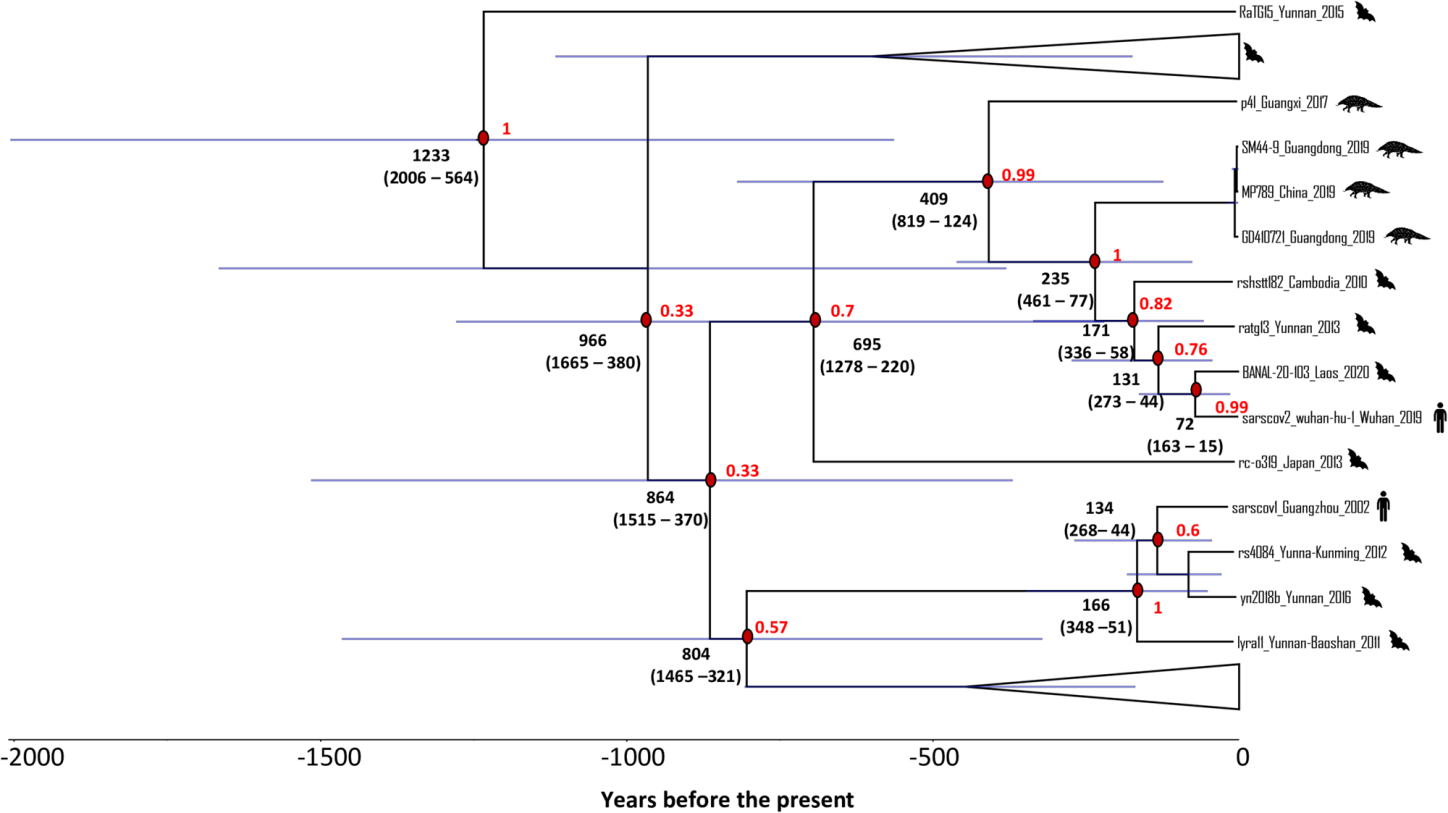
Maximum clade credibility tree of the RBD of 45 *Sarbecoviruses*. Median ages are shown in black with the 95% HPDI in brackets and represented by the error bars. Some nodes have been collapsed (triangles) to help with the visualization of the tree. Posterior node support is indicated with red. Branch lengths are scaled in time.

The clonal frame shows BANAL-103, as the closest relative of SARS-CoV-2, with RaTG13 and three pangolin Sarbecoviruses (including GD410721), representing the second and third closest lineages to the human virus respectively (Fig. 5). The root of the tree has an estimated median age of 1437 years before the present (yBP) (95% HPDI: 2020-824 yBP). The split between SARS-CoV-2 and BANAL-103 occurred around 97 yBP (95% HPDI: 159-44 yBP), while the divergence between the RBD of these viruses and RaTG13 happened 142 yBP (95% HPDI: 230-66 yBP). The common ancestor of the viruses capable of recognizing ACE2 receptors (SARS-CoV-2, BANAL-103 and GD410721), and RaTG13 has a median age of 255 yBP (95% HPDI: 398-127 yBP). A median evolutionary rate of 3.89E^−4^ substitutions/site/year (95% HPDI: 2.3E^−4^–6.73E^−4^) was estimated by Bacter. To compare phylogenies generated assuming non-recombinant and recombinant evolution a molecular dating analysis was performed in BEAST2 using a relaxed molecular clock. The maximum clade credibility (MCC) tree has a younger root with a median age of 1233 yBP (95% HPDI: 2006-564 yBP). Younger median estimates were also obtained for the internal nodes of the tree (Fig. 6). The evolutionary rate had a median estimate of 5.32E^−4^ substitutions/site/year (95% HPDI: 2.51E^−4^–1.14E^−3^). Overall, the HPDI for the node ages and the evolutionary rate estimated in both analyses greatly overlap.

While the position in both phylogenies of RaTG13 did not change, a few topological differences can be observed between the clonal frame and the MCC tree. The Sarbecovirus rshstt182 appears in the MCC tree in a closer position to SARS-CoV-2 than the pangolin viruses even if the latter ones possess a more similar RBD to the one from the human virus. Another difference is the position of the clade that contains SARS-CoV-1 and rs4084. In the clonal frame, this group of viruses and the virus rc-o319 share a most recent common ancestor with the clade that contains SARS-CoV-2. In the MCC tree on the other hand, the SARS-CoV-1 clade shares a most recent common ancestor with bat Sarbecoviruses located in the lower part of the tree. Both rshstt182 and rs4084 were involved in recombination events.

## Discussion

In this study we conducted a recombination-aware phylogenetic analysis of the RBD region of 45 *Sarbecoviruses*. Multiple recombination events with a posterior probability support greater than 0.9 involving different *Rhinolophus* species were detected, which suggests a close interaction between bat populations. In this regard, *R. sinicus*, *R. pusillus* and *R. affinis* have overlapping geographical ranges, and the last two have been proposed as the likely host of SARS-CoV-2 progenitors^36^. Recombination events #1 and #2 affected the second half of the RBD. This region contains the receptor binding motif that interacts with the cellular receptors^37^. Thus, these recombination events could have implications on host specificity and virulence. The third (Q493), fifth (N501) and sixth (Y505) residues of SARS-CoV-2 form hydrogen bonds with the hACE2 residues K31, K353 and E37, respectively, with the first two being recognized as virus-binding hot spots^11,38^. The remaining SARS-CoV-2 residues (L455, F486, and S494) enhance the binding of the first three by forming favorable interactions with hot spots 31 and 353. Different amino acid residues in the key six positions in human and civet SARS-CoV-1 have shown a diminished affinity for the human residues^11^. The same occurs in RaTG13. In the bat virus, the amino acid of residue 486 has a L (leucine) instead of a F(phenylalanine) which doesn’t fit in the binding site where F486 is inserted during hACE2 binding^39^. Residue 494 has an R (arginine) instead of an S (serine). Arginine is the most hydrophilic of all amino acids^40^, thus, this change causes R494 to be attracted to water molecules, away from the hydrophobic environments of the ACE2 binding sites^41^. The amino acid of residue 493 in RaTG13 is not a Q (glycine) but a Y (tyrosine) which cannot form hydrogen bonds with K31^39^. Residue 505 has an H (histidine) in the bat virus which makes the RBD structure less stable than in SARS-CoV-2^39^. Laboratory studies that exchanged the amino acids between SARS-CoV-2 and RaTG13 in positions 486 and 505, found a two- to five-fold reduction in infectivity in the human virus. An even greater effect was observed when the substitution N501D was inserted in SARS-CoV-2, resulting in a 100-fold decrease in infectivity. Introducing the substitutions D501N and H505Y in RaTG13 increased the infectivity of the bat virus in a similar degree^42^. The other recombinations involved the first half of the RBD, which contains epitopes or antigenic determinants that are involved in the immune response^43^. Hence, it is possible that these recombination events could have provided the virus with an enhanced capacity to escape the immune system of the host.

The presence of a significant recombination event within the RBD involving RaTG13 and the results of the ASR analysis support a non-recombinant origin for the variable loop within the RBD of SARS-CoV-2 and the common ancestor hypothesis, in which this bat virus lost all but one of the contact amino acid residues already present in the common ancestor of RaTG13, SARS-CoV-2, BANAL-103 and GD410721 (Fig. 4). This ancestral virus was likely a generalist pathogen capable of infecting different types of mammalian hosts, since laboratory studies have proved that SARS-CoV-2 can bind to the ACE2 receptors of cattle, cats and dogs^44^. The ability to bind to the hACE2 receptor has also been proposed as an ancestral trait of the whole *Sarbecovirus* subgenus, as the basal *Sarbecovirus* Khosta2, discovered in Russia, has shown this capacity in vitro^34^. While the acquisition of a derived RBD by RaTG13 through recombination seems like a very plausible scenario, it is still possible that the different amino acid residues emerged due to other processes like adaptation, before or after the recombination occurred. However, given that 18% of the amino acids of the variable loop are different between RaTG13 and SARS-CoV-2, the simultaneous replacement of the involved nucleotides due to a single recombination event, is a more parsimonious explanation. Laboratory studies have shown that RaTG13 has a high affinity for the ACE2 receptor of mammal hosts, like mouse and rat, that other related Sarbecovirus can’t efficiently infect. This suggests that RaTG13 has followed a different evolutionary path involving spillovers^45^, and possibly recombination events, in a distinct range of hosts.

No recombination events were detected between SARS-CoV-2 and BANAL-103. However the fact that bat *Sarbecoviruses*, able to recognize human cell receptors, are found in nature, highlights the importance of broad sampling of bats and other potential host species of coronaviruses, as any newly discovered virus could improve our understanding of the origin of SARS-CoV-2 tremendously. Our results support a natural emergence (through vertical evolution combined with a number of recombination events) of the genetic region that makes SARS-CoV-2 so successful at transmitting among humans. It is important to notice that only a small region of the genome was analyzed, and recombination breakpoints have been detected throughout the whole *Sarbecovirus* genome. Thus, recombination could still have been an important factor in the emergence of SARS-CoV-2, involving regions other than the variable loop of the RBD. In this regard, signals of recombination events involving the SARS-CoV-2 lineage have been detected on the 5′ and 3′ ends of the S gene^27^. In a recent study^18^, a recombination event was detected at the beginning of the SARS-CoV-2 RBD, associated with these viruses, however, it extended beyond the region included in our analysis. Recombination events involving other groups of coronaviruses could also be possible.

The results from the simulation analyses suggest that it is unlikely that the recombinations detected represent spurious signals due to the model assumptions of Bacter or false positives of the new implementation of ACGAnnotator. Furthermore, a molecular dating analysis assuming rate variation among lineages produced only small differences in the estimated node ages in comparison to the ones obtained with a strict clock in Bacter. On the contrary, differences in tree topology, involving the placement of recombinant viruses, between trees that consider recombinant and non-recombinant evolution were observed. These results showed the importance of accounting for recombination in phylogenetic reconstructions.In this study we present a recombination-aware phylogenetic analysis of *Sarbecoviruses*, which sheds light on the evolutionary origin of the variable loop in the RBD of SARS-CoV-2. Our simultaneous estimation of the vertical (tree-like) and horizontal (recombination) evolutionary history of the virus is in stark contrast to the more traditional approach that consists in the initial detection of recombination breakpoints followed by the phylogenetic reconstruction of each region located between breakpoints. While we recognize that the computational requirements of the employed approach restricted the scope of this study, as we couldn’t analyze the full data set and only analyzed a small fragment of the *Sarbecovirus* genome, we believe that the results obtained here provide an important “in-depth look” into the recombination history of the RBD. Further methodological developments could allow the analysis of full genes or even full genomes, which in turn will provide us with a more detailed overview of the role of recombination in the evolutionary history of coronaviruses and other pathogens.

## Methods

### Dataset

The dataset consisted of 111 genomes downloaded from the GenBank and GISAID databases (Supplementary Table S1 online). These included one SARS-CoV-2 sequence sampled in Wuhan in 2019, one SARS-CoV-1 sequence, thirteen *Sarbecovirus* sequences from pangolins, three from civets, and 93 from bats. The sequences were aligned with MAFFT 7.475 using the default settings^46^, producing a sequence alignment with 31,237 sites. The program Gblocks^28^ was then used to detect poorly aligned positions, which were replaced by Ns. A column of the alignment was masked if it contained at least 50% of gaps. Neighboring sites with more than 15% of gaps were also masked. The region composed by the RBD was extracted from the full genome alignment using the genomic locations of the SARS-CoV-2 reference genome NC_045512.2 to produce an RBD alignment with a length of 744 sites.

### Substitution model and tree prior selection

The program bModelTest^31^ implemented in the platform BEAST2 V2.6.3^47^ was used to select the best substitution model for the data set. The analysis ran for 200 million states. Next, a model comparison between the constant population size and the Bayesian skyline coalescent tree priors, was done with a Stepping-Stone sampling^33^. This comparison ran for 50 steps with a Markov Chain Monte Carlo (MCMC) length of 10,000,000 each, using the selected model of nucleotide substitutions. The constant coalescent was selected as the best model (logBF = 2.14).

### Test for temporal signal

The evaluation of temporal signal for the alignment was done with the BETS approach^30^. In this method, the marginal likelihoods of a heterochronous model, with the real dates (M1), and an isochronous model, with all dates set to zero and a fixed molecular clock rate (M2), are approximated in a Stepping-Stone sampling^33^. A positive log Bayes Factor value (log marginal likelihood M1–log marginal likelihood M2) indicates the presence of temporal signal. The Path Sampling analysis ran for 50 steps with a Markov Chain Monte Carlo (MCMC) length of 10,000,000 each, using the best substitution model and tree prior.

### Bayesian recombination analysis

The Bayesian recombination analysis of the RBD region was performed with Bacter, a BEAST2 V2.6.3 package that generates a sample of Ancestral Conversion Graphs (ACGs) from the posterior distribution. An ARG consists of a backbone phylogeny, known as the Clonal Frame (*C*) that represents the true genealogy of the sequences, and a set of recombinant edges (*R*) that connect two branches of the tree and represent recombination events^24^ (Supplementary Fig. S1). A Bacter analysis can be computationally demanding, and initial analyses using the 111 sequences showed that it was not feasible to analyze the whole data set, given the time and amount of resources needed. Hence, a sample of the data set was generated with Uclust^29^, and algorithm that generates clusters of sequences according to a certain threshold of genetic diversity. The cluster was selected using the *cluster_fast* command and a threshold of 96%, which represents the similarity between SARS-CoV-2 and RaTG13. 45 sequences were selected, including the SARS-CoV-2 genome and its closest relatives (RaTG13, GD410721 and one cave bat virus).

In order to improve the mixing and convergence of the analyses, Bacter was used in combination with the Metropolis coupled Markov chain Monte Carlo algorithm (MC^3^). In this extension of the traditional MCMC, multiple parallel chains are used to sample the posterior. One, known as the cold chain, explores the posterior like in the regular MCMC. The other runs are known as the “heated” chains and add an exponent β to the posterior probability. Values of β lower than one, reduce or “melt” the peaks of probability making easier the movement of the chains between regions of high probability. The cold and heated chains can exchange locations, reducing the risk of the analysis getting stuck at a local optimum^48^. In BEAST2 V2.6.3 the MC^3^ method is implemented in the CoupledMCMC package^49^.

Two independent analyses were done, each using 8 chains (7 heated) with a length of 500 million states. The constant coalescent tree prior was used, as well as the substitution model selected by bModelTest with the addition of 8 gamma rate categories to account for rate heterogeneity among sites. We also set an upper bound of 10 recombination events allowed per sampled ACG, as it is a practical solution to set an upper bound to reduce the run time of the analysis. For the prior distribution of the clock rate, a normal distribution (mean = 7.8E^−4^, σ = 3.0E^−4^) was used, similar to one previously employed in a molecular dating analysis of *Sarbecoviruses*^10^. A normal distribution (mean = 150, σ = 50) was employed as a prior for the mean length of the recombinant part of the sequence. A uniform distribution between 0 and 100,000 was used for the prior of the population size. Lastly a uniform distribution between 0 and 15,000 years was also set as the prior for the age of the root of the clonal frame, considering the results of a previous dating analysis of *Sarbecoviruses*^10^. After assessing convergence of the chains the output files were combined with the software LogCombiner^50^, removing a 10% burn-in. This yielded effective sample sizes above the standard threshold of 200 for all parameters, as assessed by the software Tracer v.1.7^51^.

A summary ACG was obtained with the ACGAnnotator tool included in the Bacter package, using a posterior support threshold of 0.5 for the recombination events. The summary method employed by Bacter merges recombinations if they connect the same pair of branches and if the recombinant region overlaps at least by 1 base pair^24^. However, there are cases where multiple recombinations arrive at the same branch but their origin points are in different locations of the clonal frame (e.g. just before or after an internal node). While Bacter considers these recombinations as independent events, it is possible that in reality they are the same recombinations, with the different origin points representing the uncertainty in the identity of the donor branch. To circumvent this problem, we also used a modified version of the ACGAnnotator code that merges recombinations with different origin points if they (1) arrive at the same branch and (2) have overlapping recombinant regions.

To test if the affinity for the ACE2 receptor given by the presence of the six contact residues is an ancestral trait within the SARS-CoV-2 clade, we performed an ancestral sequence reconstruction analysis implemented in the BEAST2 package beast-classic. The analysis ran for a MCMC of 500 million steps using the same setup from the Bacter analyses but without the MC^3^ approach which was not supported by the package.

### Simulations to test the effect of rate variation on recombination detection

A molecular dating analyses was done in BEAST2 using similar parameters as in the Bacter analyses but partitioning the alignment into three regions: (1) before first recombination breakpoint, (2) recombinant region determined by the 95%HPDI for the start and end site of the recombination affecting RaTG13, (3) after second breakpoint. For each partition an uncorrelated lognormal relaxed molecular clock model was implemented with a normal prior for the mean rate (mean = 7.8E^−4^, σ = 7.5.0E^−4^). Three replicate analyses were realized and after assessing convergence, the replicates were combined with LogCombiner. The mean and standard deviation estimates of the evolutionary rates were used to simulate 10 sequence alignments for each partition with the BEAST2 package feast. Trios of alignments were then concatenated with the software Concatenator^52^ to produce 10 alignments with the same length and number of sequences as the alignment of real sequences. The alignments were analyzed with Bacter fixing the recombination rate to the one estimated with real data (7.296E^−7^) and summary ACGs were generated with a threshold of 0.1 using the classic and new implementations of ACGAnnotator.

### Comparison of recombinant and non-recombinant evolution

Additional dating analyses without alignment partitions were done using the same parameter setup as in the Bacter analyses but assuming a relaxed molecular clock model. Two replicate analyses were done and like before, Tracer was used to assess the convergence of the analyses and LogCombiner to combine the output files. The maximum clade credibility trees of the molecular dating and the Bacter clonal frame were generated with TreeAnnotator^53^ with a 10% burn-in.

### Supplementary Information

Supplementary Figures.

Supplementary Table S1.

## Data Availability

The authors declare that all data supporting the findings of this study are available within the paper, the Supplementary Information and in publicly available data repositories. Multiple sequence alignment, xml, combined log and tree files, and simulation results, are available at https://github.com/tidelab/Sarbecovirus_recombination. Note that 26 sequences fall under the terms of use of the GISAID platform. The Bacter code with the new implementation of ACGAnnotaror tool is available at https://github.com/tidelab/bacter/tree/modify_summary_method.
