# Supplementary material for: Recombination-aware phylogenetic analysis sheds light on the evolutionary origin of SARS-CoV-2
Source: Sci Rep. 2024 Jan 4;14:541. doi: 10.1038/s41598-023-50952-1 (PMC10766966; doi:10.1038/s41598-023-50952-1)
Supplement: Supplementary file 1 — Supplementary Figures. [file 41598_2023_50952_MOESM1_ESM.pdf]

# Supplementary information

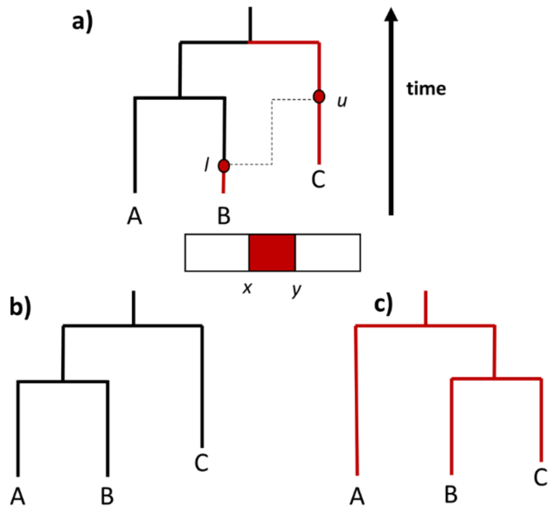


Supplementary Fig. S1. Example of an ACG of three taxa. a) A recombination event occurred at time *l* (arrival point) between lineage B and an unsampled descendant of lineage C, who emerge at time *u* (departure point). This resulted in the transfer of the red part of the genome (delimited by x and y) from C to B. b) Phylogenetic tree of the three taxa excluding the recombinant region. c) Phylogenetic tree constructed with the recombinant region, showing the conflicting topologies introduced by recombination.

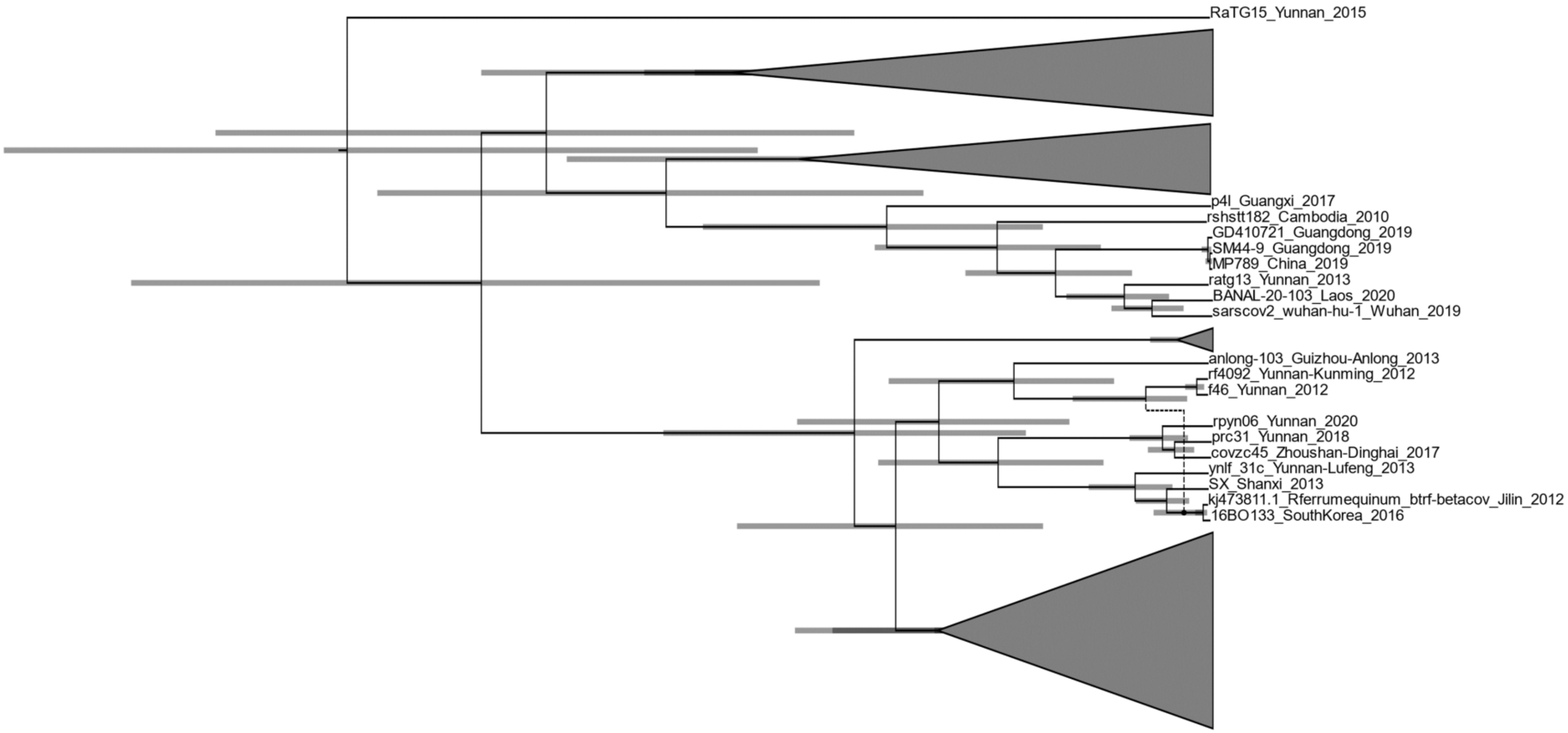


Supplementary Fig. S2. Summary ACG obtained for 45 *Sarbecovirus* sequences with a posterior threshold of 0.9 and using the original implementation of ACGAnnotator. Recombination events indicated with the dashed lines.

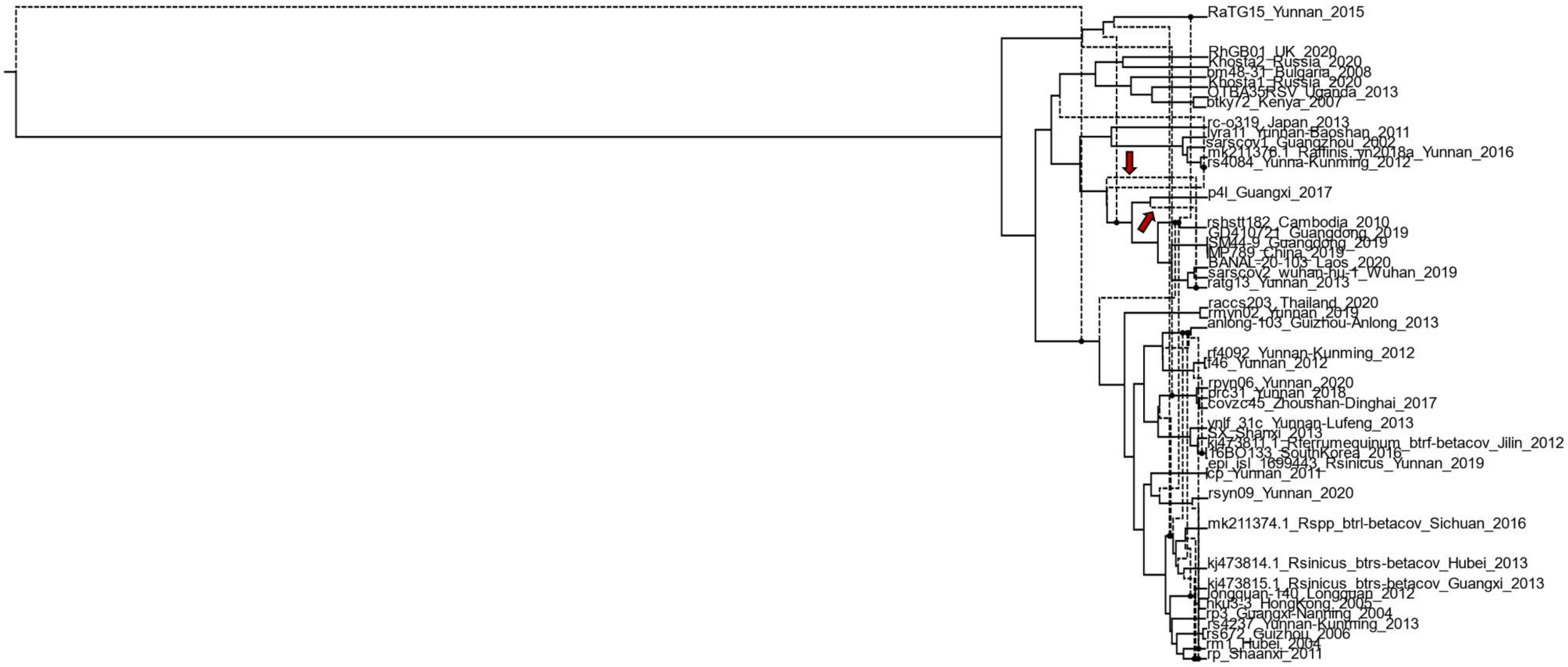


Supplementary Fig. S3. Summary ACG obtained for 45 *Sarbecovirus* sequences with a posterior threshold of 0.1 and using the original implementation of ACGAnnotator. Recombination events indicated with the dashed lines. Red arrows indicate recombination events affecting RaTG13 with different arrival points.
